# Investigating the effects of hydrostatic pressure on the physical properties of cubic Sr_3_BCl_3_ (B = As, Sb) for improved optoelectronic applications: A DFT study

**DOI:** 10.1016/j.heliyon.2024.e35855

**Published:** 2024-08-06

**Authors:** Asif Hosen

**Affiliations:** Department of Materials Science and Engineering, Khulna University of Engineering & Technology (KUET), Khulna, 9203, Bangladesh

**Keywords:** First-principles study, A_3_BX_3_ perovskite, Pressure effect, Optoelectronic properties, Mechanical properties

## Abstract

This article explores changes in the structural, electronic, elastic, and optical properties of the novel cubic Sr_3_BCl_3_ (B = As, Sb) with increasing pressure. This research aims to decrease the electronic band gap of Sr_3_BCl_3_ (B = As, Sb) by applying pressure, with the objective of enhancing the optical properties and evaluating the potential of these compounds for use in optoelectronic applications. It has been revealed that both the lattice parameter and cell volume exhibit a declining pattern as pressure increases. At ambient pressure, analysis of the band structure revealed that both Sr_3_AsCl_3_ and Sr_3_SbCl_3_ are direct band gap semiconductors. With increasing pressure up to 25 GPa the electronic band gap of Sr_3_AsCl_3_ (Sr_3_SbCl_3_) reduces from 1.70 (1.72) eV to 0.35 (0.10) eV. However, applying hydrostatic pressure enables the attainment of optimal bandgaps for Sr_3_AsCl_3_ and Sr_3_SbCl_3_, offering theoretical backing for the adjustment of Sr_3_BCl_3_ (B = As, Sb) perovskite's bandgaps. The electron and hole effective masses in this perovskite exhibit a gradual decrease as pressure rises from 0 to 25 GPa, promoting the conductivity of both electrons and holes. The elastic properties are calculated using the Thermo-PW tool, revealing that they are anisotropic, ductile, mechanically stable, and resistant to plastic deformation. Importantly, these mechanical properties of both compounds are significantly enhanced under pressure. Optical properties, including the absorption and extinction coefficients, dielectric function, refractive index, reflectivity, and loss function, were calculated within the 0–20 eV range under different pressure conditions. The calculated optical properties highlight the versatility and suitability of Sr_3_AsCl_3_ and Sr_3_SbCl_3_ perovskites for pressure-tunable optoelectronic devices.

## 1 Introduction

The creation of efficient devices necessitates the use of physically stable and high-quality materials. A broad spectrum of materials is available for selection, but their application in technical progress is limited by physical constraints. Material scientists are currently focusing their attention on developing low-cost materials with the ability to maintain their properties under extreme conditions. In such a scenario, perovskite compounds may be extremely important. In recent years, researchers have directed their focus toward cubic perovskite compounds due to their versatility in various branches, encompassing sensors, photovoltaic cells, LEDs, superconductivity, photoelectric cell-containing devices, and semiconductors [[1], [2], [3]]. These perovskite materials are appropriate for creating nanocrystals, nanowires, and nanoparticles, and they can be modified to accommodate different structures [[4], [5], [6]]. Consequently, technology relying on perovskite compounds is anticipated to be more economical and suitable compared to silicon-based technology [7]. The general expression for cubic perovskite materials is identified as ABX_3_, with A denotes a monovalent cation, B represents a bivalent metallic cation, and halogen anions occupy the X-position. Solar cells based on perovskite have garnered notable recent attention, surpassing several established thin-film solar cell devices with power conversion efficiencies surpassing 15 %, with a focus on methylammonium lead trihalide perovskites in much of the research [8]. However, most perovskite halides with exceptional properties contain lead (Pb), presenting two significant challenges: the chemical instability and toxicity of lead, which could have adverse impacts on both human well-being and the surrounding ecosystem. Due to environmental contamination and the global energy crisis, there has been considerable interest in clean and sustainable energy sources. Given these conditions, the European Union and other countries have imposed stringent limitations on the use of lead-based perovskites in optoelectronic devices, resulting in an acceleration of investigation on Pb-free Perovskite Solar Cells (PSCs) [[9], [10], [11]]. Therefore, there is a significant demand in the solar cell industry to investigate lead-free and high-efficiency photovoltaic materials with a focus on commercialization. Consequently, recently, a significant amount of experimental and theoretical work has been conducted, focusing on substituting lead with an appropriate metal cation [[12], [13], [14], [15]]. The lead-free halide perovskites GaBeCl_3_ and GaCaCl_3_ exhibit direct band gaps [16], making them promising for use in optoelectronic devices and as insulating materials. Additionally, an alternative method to tackle this issue involves substituting lead-based perovskites with lead-free halide double perovskites, which have shown promising optoelectronic applications and excellent stability, featuring both smaller and larger bandgaps [[17], [18], [19]].

First-principles calculation was effectively implemented for perovskite materials to analyze diverse physical attributes [20]. The physical attributes of perovskite materials can be changed through substituting elements [21], doping [22,23], or applying hydrostatic pressure [[24], [25], [26]]. Linh et al. [27] discovered that substituting alkali metals with larger ionic radii (M = Li, Na, and K) in (Bi_0.5_M_0.5_) TiO_3_ resulted in a higher electronic band gap. Gillani et al. [22] documented that incorporating alkaline earth elements (Mg, Ca, Ba) into SrZrO_3_ caused a shift from an indirect band gap to a direct band gap. The implementation of hydrostatic pressure has the capability to selectively modify the physical characteristics of both A_3_BX_3_ and ABX_3_ perovskite materials. This approach has proven successful in tailoring perovskite materials to meet specific technological needs. Using this technology, scientists can precisely adjust the physical properties of cubic perovskite materials. Pressure serves as a clean tuning mechanism for modifying crystal arrangement and their electrical characteristics without altering the chemical composition [28,29]. Besides regular physical attributes, the pressure-dependent opto-electronic properties of perovskite materials have also been documented [30,31]. Perovskite materials subjected to pressure can be employed in p-n junctions, multiple quantum wells, heterostructures, and various sectors such as photovoltaics and optoelectronic devices [32,33]. Babu et al. [34]investigated the CsSnCl_3_ perovskite material and observed a decrease in the band gap, resulting in a transformation from a semiconductor to a metallic phase when a compressive force is implemented.

In photovoltaic technology, inorganic compounds have become notable because of their wide range of physical properties. In recent times, numerous investigations [[35], [36], [37], [38], [39]] have scrutinized various inorganic compounds denoted as A_3_BX_3_, with A represents a larger-sized inorganic cation, B denotes a smaller metal cation, and X signifies an anion. Considering the essential screening criteria including appropriate bandgap, high optical absorption coefficient, theoretical PCEs (>25 %), thermal stability, and significant band edge transition probabilities (P^2^), Ba_3_PI_3_, Ba_3_AsI_3_, and Ba_3_SbI_3_ are forecasted to serve as most stable solar cell candidates, demonstrating an efficiency surpassing 25 % [39]. Islam et al. [40] studied the physical properties of Ca_3_AsBr_3_ and discovered that the bandgap of this compound could be altered through the application of hydrostatic stress, leading to a shift from semiconductor to metallic behavior at 50 GPa. Rahman et al. [41] recently conducted a theoretical study on how strain affects the structural, and optoelectronic properties of the lead-free cubic perovskite Sr_3_AsCl_3_. Their research uncovered that the cubic Sr_3_AsCl_3_ material possesses a direct bandgap, making it highly suitable for applications in solar cells for both energy generation and light control. Furthermore, the bandgap of Sr_3_AsCl_3_ shows a decline due to implementation compressional strain and a rise under tensional strain. However, the mechanical behavior of Sr_3_AsCl_3_ under both stressed and unstressed conditions remain unknown. Also, as far as we are aware, there has been no comprehensive research conducted on the electronic, mechanical, and optical characteristics of Ca_3_SbCl_3_ under both pressurized and unpressurized conditions. Therefore, it is imperative to uncover the physical properties of the Sr_3_BCl_3_ (B = As, Sb) compound under high pressure because various alterations in physical phenomena, including phase transformation and modification of the physical and chemical attributes of compounds, can occur at elevated pressures. This has influenced us to explore the structural, mechanical, and optoelectronic characteristics of Sr_3_BCl_3_ (B = As, Sb) within a hypothetical perovskite framework under pressured conditions.

Both Sr_3_AsCl_3_ and Sr_3_SbCl_3_ perovskite compounds belong to the category of perovskites [39], and their cubic phase has been theoretically verified by Born stability criterion. Our primary aim is to clarify how Sr_3_BCl_3_ (B = As, Sb) responds to applied pressure. Sr_3_BCl_3_ (B = As, Sb) is a perovskite material recognized for its non-toxicity, and this study aims to improve its optoelectronic and mechanical attributes. Applying compressional force is intended to refine its crystal structure and enhance carrier mobility, which should enhance the material's charge transport properties and increase its efficiency as a photovoltaic material. Moreover, applying pressure can significantly enhance the optical properties of halide perovskites, thereby advancing their functionality in optoelectronic applications. Before incurring exorbitant experimental costs, utilizing efficient and cost-effective software to theoretically explore such novel materials is a practical approach. Hence, this research is centered on theoretically investigating the physical properties of novel Sr_3_BCl_3_ (B = As, Sb) perovskite for potential use in optoelectronic devices in both ground and pressure-induced conditions.

## 2 Computational details

The first-principles computation was conducted utilizing the Quantum Espresso simulation application software [[42], [43], [44]], which implements the plane-wave pseudo-potential total energy method grounded in density functional theory (DFT). A norm-conserving (NC) pseudopotential [45,46]and the Perdew-Burke-Ernzerhof (PBE) [47] exchange-correlation mechanism was employed in the FP-DFT study of the Sr_3_BCl_3_ (B = As, Sb) perovskite structure. Because DFT-PBE calculations notably underestimate the bandgap in comparison to experimental measurements, the Heyd-Scusena Ernzerhof (HSE) method [48] was utilized to precisely determine the bandgap, while all other calculations were conducted using the GGA-PBE functional. The wavefunction cutoff, Monkhorst–Pack k-point grid, and lattice parameter were optimized by computing and minimizing their energies across various values. Improvements in simulation and structural optimization performance were achieved by implementing kinetic energy cut-off and charge density cut-off values fixed at 40 Ry and 440 Ry, respectively. The Broyden-Fletcher-Goldfarb-Shanno (BFGS) minimization approach [49], which is renowned for its effectiveness in discovering the structure with the lowest energy, was utilized for geometry optimization. In the process of structural optimization, the hydrostatic pressure was gradually increased from 0 to 25 GPa, with increments of 5 GPa. The most suitable crystal structure was produced utilizing the VESTA program [50]. For Self-Consistent Field (SCF) calculations, the k-points (k_x_, k_y_, k_z_) were setup as 8 × 8× 8, while for Non-Self-Consistent Field (NSCF) computations, they were set up as 12× 12× 12. In the Self-Consistent Field (SCF) computations, a maximum force tolerance of below 0.01 eV/Å and a convergence threshold of 10^−6^ a.u. were implemented. The force convergence threshold was set up as 10^−3^ a.u. for the ionic minimization in relaxation calculations. The assessment of material structures dynamical stability and the exploration of their optical behavior were examined by utilizing the first-order time-dependent perturbation theory [51]. After that, the absorption peaks of the complex dielectric function were identified through an analysis of the photon energy spectrum. It is recognized that the key equation for calculating optical absorption coefficients is the complex dielectric function, determined with the equation [52], ε(ω) = ${\;\varepsilon }_{1}\left(\omega \right)$ + $i\varepsilon_{2}\left(\omega \right)$. The Thermo-PW package was employed for the computation of the mechanical properties. Researchers [53,54] use the "Thermo_PW" module in Quantum Espresso by setting up appropriate input files that define the crystal structure, and the range of strains to apply. The elastic moduli were determined using the Voigt–Reuss–Hill approximation (VRH) [55], which calculates the average of the Voigt and Reuss approximations.

## 3 Results and discussions

### 3.1 Structural properties

**Fig. 1:**
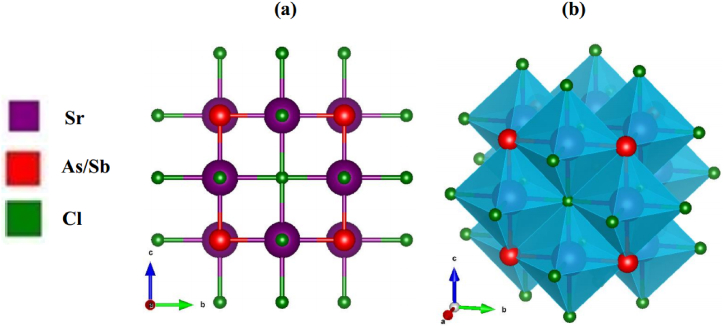
Optimized crystal structure (2D and 3D view) of Sr_3_BCl_3_ (B = As, Sb).

**Fig. 2:**
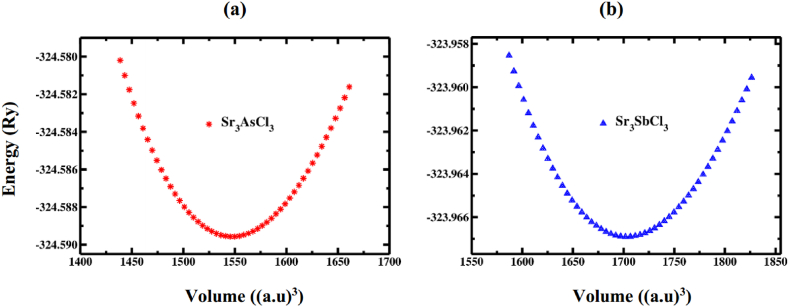
Total energies against volume curve for Sr_3_BCl_3_ (B = As, Sb).

Initially, a comprehensive optimization of the structure of strontium-based novel perovskite Sr_3_BCl_3_ (B = As, Sb) was conducted. The relaxed optimized structures (both 2D and 3D visualization) are illustrated in Fig. 1a and b. Both structures exhibit cubic symmetry and fall within the space group pm-$\bar{3}$ m (221) [39]. These structures are limited to unit cells containing a maximum of seven atoms. The Wyckoff notation, utilized to depict equivalent positions within the unit cell, can represent the fractional coordinates of Sr, As (Sb), and Cl atoms within Sr_3_BCl_3_ (B = As, Sb). The Sr cations were positioned at Wyckoff coordinates of 3d (0, 0.5, 0) and the As/Sb cations at 1a (0, 0, 0). Furthermore, the positions of the three Cl anions are specified as 3c (0.5, 0, 0.5), (0.5, 0.5, 0), and (0, 0.5, 0.5), respectively. The structure parameters, including the lattice constant (*a*_0_) and the ground state energy (*E*_0_), were computed using the Birche-Muranghan equation [56]. The Bulk modulus (*B*_0_), which signifies the material's hardness, along with its derivative (*B*^/^), was also acquired. All the structural properties that were computed are detailed in Table 1. The stability of the investigated compound's structures was also determined by analyzing the energy plotted against volume curve, as illustrated in Fig. 2a and b. The optimized lattice constants (Table 1) under ambient pressure for Sr_3_AsCl_3_ and Sr_3_SbCl_3_, as determined in this research, align precisely with those obtained in Ref. [39], confirming the precision of our calculations. Furthermore, the enthalpy of formation is determined using the formula provided below.

$$E_{f}\left({\text{Sr}}_{3}B{\text{Cl}}_{3}\right)=\frac{\left[E_{\text{tot}.}\left({\text{Sr}}_{3}B{\text{Cl}}_{3}\right)-3E_{s}\left(\text{Sr}\right)-E_{s}\left(B\right)-3E_{s}\left(\text{Cl}\right)\right]}{N}$$

**Table 1:** Structural parameters of the unit cell and band gap of novel perovskites Sr_3_BCl_3_ (B = As and Sb).

Optimized Structural Parameters	Sr_3_AsCl_3_		Sr_3_SbCl_3_	
	Present Work	Other Cal.	Present Work	Other Cal.
Lattice constant (a_0_) in Å	6.12	6.12 [39,41]	6.32	6.32 [39]
Optimum volume V_0_ in Å^3^	229.22		252.43	
Bulk modulus (*B*) in GPa	31.6		27.3	
Pressure derivative of bulk modulus *B*′	4.34		4.25	
Ground state energy *E*_0_ in eV	− 4416.26		− 4407.79	
Band Gap *E*_g_ (Γ- Γ) in eV	1.70^PBE^	1.65^PBE^ [41]	1.72^PBE^	2.44^HSE^ [39]
	2.47^HSE^	2.47^HSE^ [39]		
Formation energy (eV/atom)	− 4.40		− 4.17

**Fig. 3:**
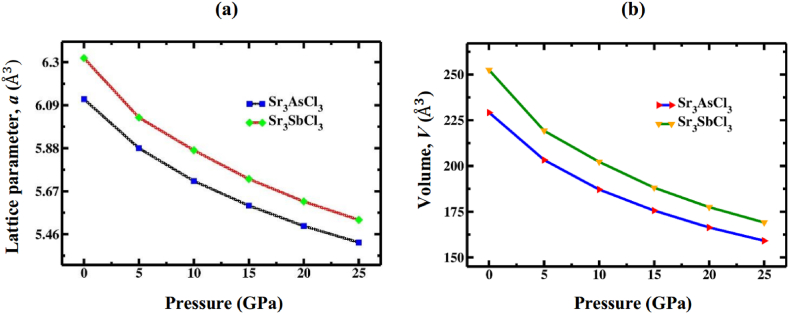
Variation of (a) lattice constant, and (b) volume for Sr_3_BCl_3_ (B = As, Sb) against pressure.

Negative formation energies (Table 1) indicate that SrAsCl_3_ and Sr3SbCl_3_ are energetically stable and viable for experimental production. With increasing the levels of compressive stress, the lattice constant and volume of Sr_3_AsCl_3_ and Sr_3_SbCl_3_ both decrease as interatomic distance is reduced (Table 2). Fig. 3a and b shows alternation in lattice constant and unit cell volume up to 25 GPa pressure, respectively. The bond lengths (Sr–Cl, Sb (As)–Cl and Sr–As (Sb)) shorten with increasing pressure levels as expected, as seen in Table 3.

**Table 2:** Calculated value of lattice constant (*a*) and unit cell volume (*V*) of Sr_3_AsCl_3_ and Sr_3_SbCl_3_ perovskites at variant pressure 0–25 GPa.

Phase	Compound	Calculated data	Pressure (GPa)					
			0	5	10	15	20	25
Cubic (pm $\bar{3}$ m)	Sr_3_AsCl_3_	*a* (Å)	6.12	5.88	5.72	5.60	5.50	5.42
		*V* (Å^3^)	229.22	203.30	187.15	175.62	166.37	159.22
	Sr_3_SbCl_3_	*a* (Å)	6.32	6.03	5.87	5.73	5.62	5.53
		*V* (Å^3^)	252.43	219.25	202.26	188.13	177.50	169.11

**Table 3:** Calculated bond length in Sr_3_AsCl_3_ and Sr_3_SbCl_3_ cubic perovskites under various hydrostatic pressures.

Pressure (GPa)	Bond length (Å)					
	Sr_3_AsCl_3_			Sr_3_SbCl_3_		
	Sr–Cl	As–Cl	Sr–As	Sr–Cl	Sb–Cl	Sr–Sb
0	3.060	4.327	3.060	3.160	4.469	3.160
5	2.938	4.155	2.938	3.018	4.268	3.018
10	2.858	4.043	2.858	2.929	4.142	2.929
15	2.799	3.959	2.799	2.863	4.049	2.863
20	2.751	3.891	2.751	2.810	3.974	2.810
25	2.712	3.835	2.712	2.766	3.912	2.766

### 3.2 Electronic properties

**Fig. 4:**
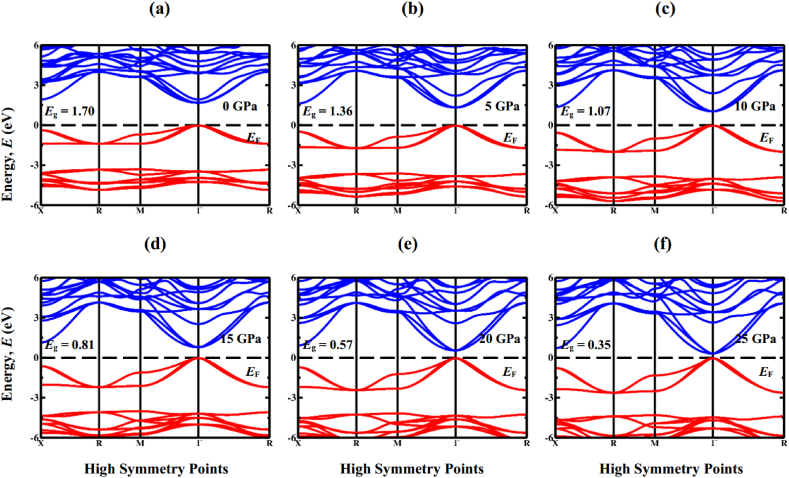
Calculated band structure of Sr_3_AsCl_3_ under various pressure (0 GPa, 5 GPa, 10 GPa, 15 GPa, 20 GPa, and 25 GPa).

**Fig. 5:**
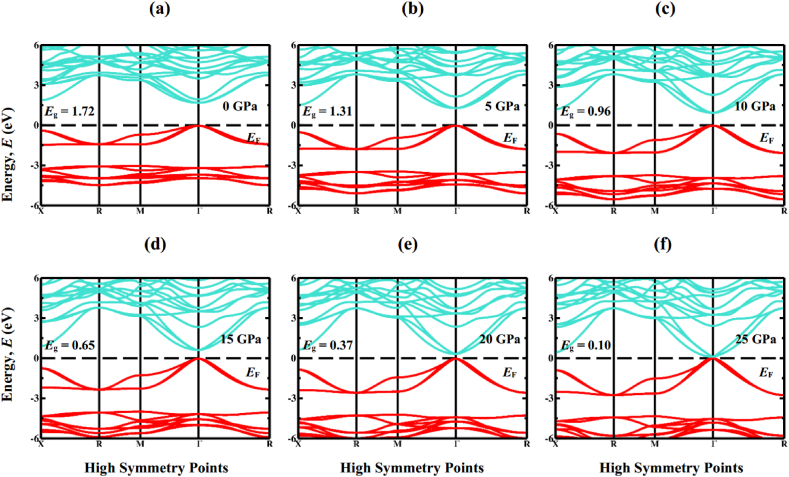
Calculated band structure of Sr_3_SbCl_3_ under various pressure (0 GPa, 5 GPa, 10 GPa, 15 GPa, 20 GPa, and 25 GPa).

**Fig. 6:**
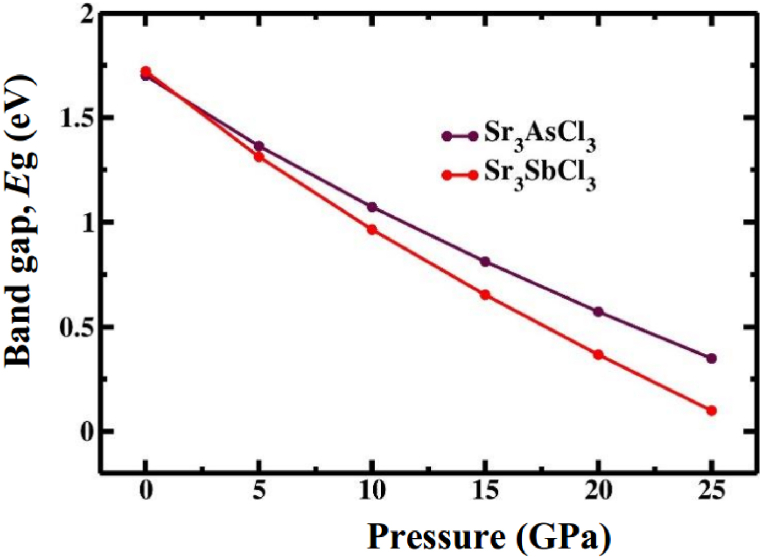
Band gaps of Sr_3_BCl_3_ (B = As, Sb) calculated using PBE method as a function of applied pressure.

Exploring the electronic properties, including electronic band structure profile and density of states (DOS), is crucial for gaining a comprehensive understanding of the optical characteristics. Determining the band structure is essential to understand whether a material has a direct or indirect band gap nature. Fig. 4(a–f) and 5 (a-f) displays the electronic band structure profiles of cubic Sr_3_BCl_3_ (B = As, Sb) perovskite under various hydrostatic pressures, as computed through GGA-PBE functional. The perovskite compounds Sr_3_BCl_3_ (B = As, Sb) exhibit unique electronic properties, as indicated by their energy band arrangement at the high-symmetry points belong to the first Brillouin zone, denoted by X − R − M − Γ− R. The effective energy associated with the principal electronic characteristics of these materials, predominantly falls within the energy span of −6 to 6 eV. Fig. 4, Fig. 5a clearly show that in the initial state, both compounds exhibit a conduction band maximum (CBM) and valence band minimum (VBM) positioned at the Γ point of the first Brillouin zone, resulting in the creation of semiconductors that exhibit direct band gap for both compounds. The computed band gaps for Sr_3_AsCl_3_ and Sr_3_SbCl_3_ are 1.70 and 1.72 when employing the GGA-PBE functional, whereas they measure 2.47 and 2.44 with the HSE approach, respectively. The heightened hybridization elevates the VBM and lowers the CBM at the Γ point of the Brillouin zone (Fig. 4, Fig. 5), resulting in a linear reduction of the band gap (Fig. 6) from 1.70 (1.72) eV to 0.35 (0.10) eV in the Sr_3_AsCl_3_ (Sr_3_SbCl_3_) perovskite. The evaluation of the bandgap clearly indicated an underestimation, a typical drawback associated with the GGA approach. Several researchers have proposed various methods, including the hybrid functional [57] and GW approaches [58], to tackle this particular discrepancy in bandgap determination. Nevertheless, each of these approaches comes with its own distinct set of limitations. In this research, the inaccuracy in bandgap estimation resulting from the GGA approach was overlooked, as the study solely concentrated on investigating the influence of hydrostatically applied pressure on the bandgap of Sr_3_BCl_3_ (B = As, Sb).

The applied pressure significantly influences the energy band gap and its characteristics. The bandgap of single-junction solar cells in HC(NH_2_)_2_PbI_3_ perovskite was reduced for the first time, from 1.489 to 1.337 eV, through the modulation of the valence electron wavefunction using a relatively low hydraulic pressure reaching up to 2.1 GPa [59]. The adjustment of this band gap enhances the effectiveness of perovskite solar cells utilizing germanium by aligning their band gap values within the optimal range as suggested by the Shockley–Queisser theory. Since then, bandgap engineering has garnered significant interest. In this segment, we conducted calculations to determine the bandgaps of Sr_3_AsCl_3_ and Sr_3_SbCl_3_ under varying hydrostatic pressure, aiming to identify the optimal bandgap for solar cells. At zero pressure, Sr_3_AsCl_3_ displays a band gap of 1.70 eV, which decreases to 0.35 eV at 25 GPa. Conversely, Sr_3_SbCl_3_ has a band gap of 1.72 eV under 0 GPa and 0.10 eV under 25 GPa. The decreased band gap enables both materials to further improve its electrical conductivity, thereby facilitating faster electron movement. This change in electronic behavior holds significant potential in electronics, with applications ranging from the development of transistors to integrated circuits.

**Fig. 7:**
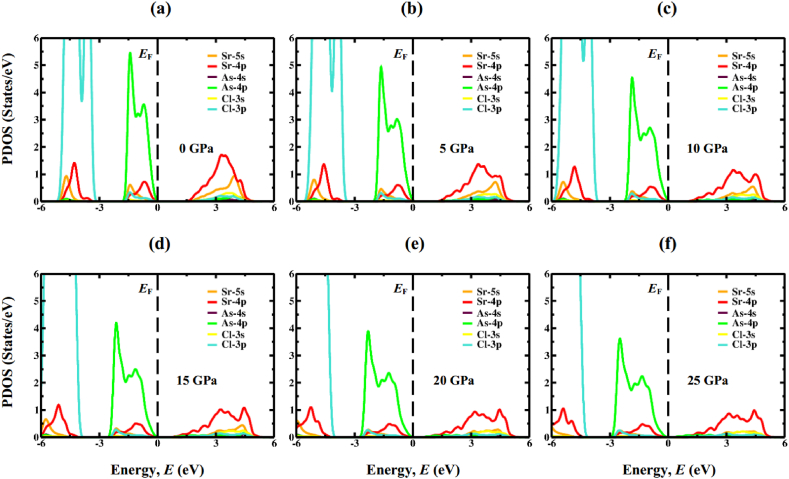
The DOS profile of cubic Sr_3_AsCl_3_ at different pressure (0 GPa, 5 GPa, 10 GPa, 15 GPa, 20 GPa, and 25 GPa).

**Fig. 8:**
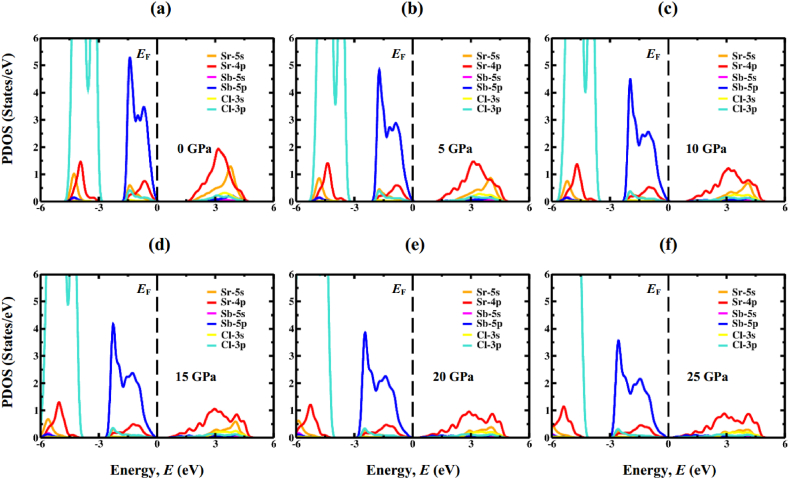
The DOS profile of cubic Sr_3_SbCl_3_ at different pressure (0 GPa, 5 GPa, 10 GPa, 15 GPa, 20 GPa, and 25 GPa).

**Fig. 9:**
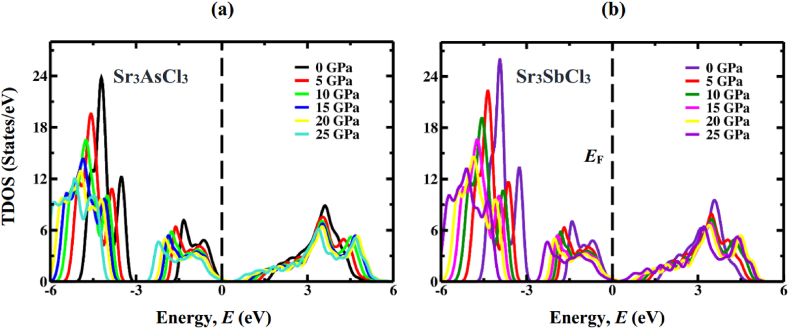
Calculated TDOS of Sr_3_BCl_3_ (B = As, Sb) under pressure.

The electronic band structures, as calculated, are then analyzed by the means of total density of states (TDOS) and partial density of states (PDOS) computations. DOS represents the participation of electronic states to the VB and CB, and thus, it is one of way to the accurate prediction of the fundamental band gap size. PDOS for Sr_3_AsCl_3_ and Sr_3_SbCl_3_ have been figured out and plotted for the energy span between −6 and 6 eV as displayed Fig. 7, Fig. 8, respectively. The region located on the left side of the Fermi energy (*E*_F_) stands for valence band, whereas the area to the right of *E*_F_ represents the conduction band. In both the non-pressurized and pressurized systems, the VB primarily arises from the As-4p (Sb-5p) state, with minimal involvement from Cl-3p and Sr-4p states. Conversely, the CB is chiefly influenced by the 4p orbital of Sr atom, with a small involvement from the Sr-5s orbital. Increasing compressive stress enhances the hybridization between As-4p (Sb-5p) and Sr-4p orbitals, shifting the CB closer to *E*_F_, consequently reducing the band gap. Furthermore, the reduction in Sr–As (Sb) bond length against compressive force (Table 3) would intensify the hybridization between the As-4p (Sb-5p) and Sr-4p states. TDOS indicates that as the amount of pressure rises, the sharp band peaks progressively migrate in the direction of high energy region closer to the *E*_F_, as shown in Fig. 9a & b. These discoveries confirm that the band gap reduction is indeed attributed to the application of hydrostatic pressure, a conclusion further supported by calculations of TDOS.

**Fig. 10:**
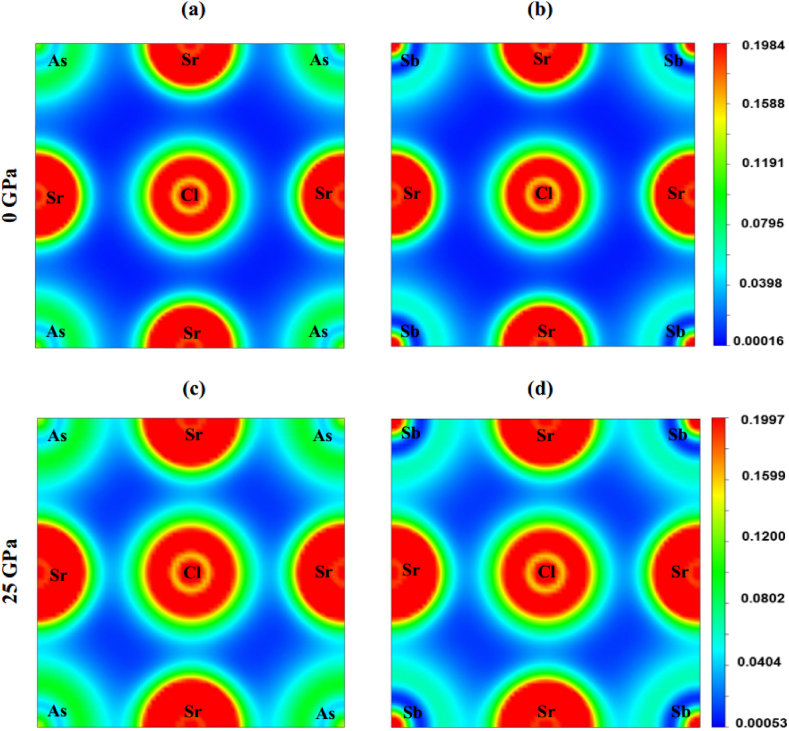
The charge density distribution of Sr_3_AsCl_3_ (a&c) and Sr_3_SbCl_3_ (b&d) at 0 and 25 GPa pressure along the crystallographic plane (100).

To acquire a deeper understanding of the chemical bonding within Sr_3_BCl_3_ (B = As, Sb), Fig. 10(a–d) depict the distribution of charge density along the crystallographic planes (100) at 0 GPa and 25 GPa applied pressure. The charge density refers to the quantity of electric charge contained within a specific region of a surface or within a particular volume of a field. It informs us about the amount of charge present within a given field. The scale positioned on the right-hand side of the density map denotes the magnitude of the charge density. It is evident from Fig. 10(a–b) that at 0 GPa, the electronic distribution surrounding Sr and Cl is nearly spherical, strongly suggesting that the bonds between Sr and Cl are predominantly ionic. However, as pressure increases, the distance between Sr and Cl diminishes along the (100) plane without causing overlap in the charge distribution, as displayed in Fig. 10c & d. On the contrary, there is a significant distortion (overlapping) in the electron distribution between Sr and As/Sb atoms at 0 GPa, indicating the existence of a covalent bond between the Sr and As/Sb atoms. The overlap between the Sr and As–Sb atoms increases further along the (100) plane at 10 GPa, thereby reinforcing the covalent character of the Sr–As and Sr–Sb bonds.

### 3.3 Carrier mobility and separation

The efficiency of a semiconductor's photocatalytic activity greatly depends on how quickly photoinduced charge carriers separate and disperse. It is commonly known that when the effective mass of the photoinduced carrier is lower, there's a higher chance that the carriers will reach the surface reaction sites during their lifespan, leading to enhanced photocatalytic activity [60]. The effective masses of Sr_3_AsCl_3_ and Sr_3_SbCl_3_ were determined by adjusting the VBM and CBM using the following formula [61] as a basis.

(1) $$m^{*}=\hbar^{2}\left[\frac{\partial^{2}\varepsilon \left(k\right)}{\partial^{2}k}\right]$$

The symbols k, ℏ, and *ε* (k) represent, respectively, the wave vectors along different axes, the reduced Planck constant, and the function describing the energy dispersion relation. Since the effective mass of both Sr_3_AsCl_3_ and Sr_3_SbCl_3_ materials falls below 1.5*m*_0_ (Table 4), it can be inferred that they can be viewed as potential candidates for solar applications requiring high mobility.

**Table 4:** Effective masses of electron and hole of Sr_3_AsCl_3_ and Sr_3_SbCl_3_ at different pressure levels. The unit of mass is the electron rest mass *m*_0_.

Pressure (GPa)	Sr_3_AsCl_3_			Sr_3_SbCl_3_		
	*m*_e_	*m*_h_	D	*m*_e_	*m*_h_	D
0	0.572	0.570	1.005	0.638	0.553	1.153
10	0.479	0.430	1.114	0.514	0.389	1.322
20	0.420	0.341	1.231	0.440	0.294	1.496
30	0.377	0.276	1.365	0.381	0.226	1.688
40	0.340	0.226	1.501	0.330	0.192	1.719
50	0.304	0.201	1.513	0.288	0.167	1.716

With increasing pressure, the effective masses of electrons and holes in Sr_3_BCl_3_ (B = As, Sb) perovskites consistently decrease, facilitating easier diffusion of electrons and holes from the absorption layer to the transport layer. This suggests that the application of hydrostatic pressure can improve the migration of carriers, thereby enhancing the PCEs of Sr_3_BCl_3_ (B = As, Sb) perovskite solar cells. As depicted in Table 4, the effective mass of holes (*m*_e_) exceeds that of electrons (*m*_h_) under all pressure conditions, indicating the n-type semiconductor properties of both compounds. The relative ratio of the effective mass (*D*), which provide knowledge about the photocatalytic characteristics of a semiconductor, is calculated using the following formula [62,63]:

(2) $$D=\frac{m_{e}}{m_{h}}$$

When the value of *D* significantly differs from 1, the discrepancy in mobility between electrons and holes becomes more noticeable, leading to a decreased recombination rate of electron-hole photo-generated pairs. The results obtained from Table 4 show that with increasing pressure, the value of *D* significantly diverges from 1 for both materials, resulting in a reduction in electron-hole recombination. From this observation, we can infer that under pressure, Sr_3_BCl_3_ (B = As, Sb) materials display a reduced effective mass and demonstrate an increased ability to separate photo-excited electron-hole pairs compared to the unpressurized system. Furthermore, Sr_3_AsCl_3_ exhibits a lower value of *D* compared to Sr_3_SbCl_3_ across all pressure ranges, thereby enhancing its suitability for photocatalytic activity.

### 3.4 Mechanical properties

The elastic constants *C*_ij_ are pivotal in establishing the structural integrity and mechanical behavior of a solid. These values indicate the extent to which a material undergoes deformation under an applied force and subsequently returns to its initial shape upon the removal of the force. can be used to observation of the material's anisotropic behavior, ductility, stability, brittleness, and stiffness. To study the mechanical properties, the finite strain theory [64] is used in this research. Since we noted a decrease in the Sr_3_BCl_3_ (B = As, Sb) lattice parameters along with increased pressure, there is a need to examine the influence of compressive stress on *C*_ij_ to gain full knowledge of the material's mechanical characteristics. The elastic properties of cubic Sr_3_BCl_3_ (B = As, Sb) can be defined using three independent elastic constants: C_11_, C_12_, and C_44_. In this work, *C*_ij_ was computed via the Thermo-PW. Table 5 presents the elastic constant values for Sr_3_BCl_3_ (B = As, Sb) at different levels of hydrostatic pressure (0–25 GPa). The Born stability criterion [65] guarantees mechanical stability, which is assessed through these sets of inequality equations:

(3) $$C_{11}>0,C_{44}>0,C_{11}-C_{12}>0,C_{11}+2C_{12}>0$$

**Table 5:** Calculated value of elastic constants (*C*_*ij*_) in GPa, Cauchy pressure (*C*_p_) in GPa, machinability index (*μ*_M_), and hardness factor (*H*_v_) in GPa of Sr_3_BCl_3_ (B = As and Sb) perovskites at variant pressure 0–25 GPa.

Pressure (GPa)	Compound	*C*_11_	*C*_12_	*C*_44_	*C*_p_	*μ*_M_	*H*_v_
0	Sr_3_AsCl_3_	75.07	10.03	15.21	− 5.18	2.08	4.16
	Sr_3_SbCl_3_	65.29	8.40	12.57	− 4.17	2.17	3.35
5	Sr_3_AsCl_3_	126.84	14.98	14.34	0.64	3.64	2.82
	Sr_3_SbCl_3_	115.97	12.95	11.66	1.29	3.62	2.78
10	Sr_3_AsCl_3_	174.26	19.48	12.75	6.73	5.57	1.98
	Sr_3_SbCl_3_	161.92	17.01	10.09	6.92	6.47	1.34
15	Sr_3_AsCl_3_	219.15	23.79	10.74	13.05	8.27	1.37
	Sr_3_SbCl_3_	205.18	20.87	8.15	12.72	10.09	0.80
20	Sr_3_AsCl_3_	262.06	27.87	8.46	19.41	12.52	0.88
	Sr_3_SbCl_3_	246.15	24.50	5.95	18.55	16.53	0.38
25	Sr_3_AsCl_3_	302.95	31.80	5.95	25.85	20.53	0.47
	Sr_3_SbCl_3_	284.39	27.73	3.48	24.25	32.55	0.01

Our findings indicate that the positive values of the determined *C*_ij_ (Table 5), coupled with their adherence to the Born stability criterion, imply that both materials maintain mechanical stability when placed under pressure conditions up to 25 GPa. The numerical value of *C*_11_ exceeds both *C*_12_ and *C*_44_ implies that the compounds under examination demonstrate resistance to compression along the x-axis [66]. Furthermore, it can be observed that the numerical value of *C*_11_ responds swiftly to pressure, *C*_12_ adapts gradually to pressure, and *C*_44_ exhibits a slight increase when stressed.

The machinability index, $\mu_{M}$, delineates a compound's cutting capacity, the highest economic level for machine operations, and the plastic strain, which is crucial in industrial sectors. It is calculated in the following manner [67]:

(4) $$\mu_{M}=\frac{B}{C_{44}}$$

Table 5 indicates that Sr_3_SbCl_3_ demonstrates a higher $\mu_{M}$ in contrast to Sr_3_AsCl_3_, indicating significantly better lubrication, reduced friction, and superior machinability, which significantly affects the manufacturing process. Furthermore, under pressure, both compounds experience a notable enhancement in machinability (Table 5), due to an increase in ductility. The rise in ductility and machinability index suggests a decline in the hardness factor.

Material hardness, another macroscopic bulk property, finds various applications in industry. A material exhibiting high hardness demonstrates greater resistance to plastic deformation when compared to one with low hardness. The common approach for assessing hardness is through the Vickers hardness ($H_{v}$), defined by Ref. [68]:

(5) Hv=2(K2G)0.585−3;K=GB

We've observed a reduction in the hardness of Sr_3_AsCl_3_ (Sr_3_SbCl_3_) under pressure, with values declining from 4.16 GPa (3.35 GPa) to 0.47 GPa (0.01 GPa) as the pressure reduces from 0 to 25 GPa (Table 5).

Evaluating mechanical parameters like shear modulus (*G*), bulk modulus (*B*), Young's modulus (*Y*), Poisson's ratio (*ν*), and Pugh's ratio (*B/G*) is important for accurately identifying the real-world uses of a material. The bulk modulus (*B*) provides an indication of a material's hardness, where a higher *B* value signifies a harder material. The stiffness of a substance is determined through its Young's modulus (*E*), where a larger values suggest increased stiffness. The shear modulus (*G*) evaluates a material's capacity to withstand plastic deformation, and a smaller value of *G* indicates less rigidity and a decreased capacity to retain its shape. The elastic moduli, including *B*, *G*, and *E*, can be determined with the Voigt–Reuss–Hill approximation (VHR). The numerical *B*, *G*, and *E* values for Sr_3_AsCl_3_ are higher compared to Sr_3_SbCl_3_ under all applied pressures (Table 6). This leads to the conclusion that Sr_3_AsCl_3_ endure more change in volume, withstands greater transverse deformation, and is stiffer than Sr_3_SbCl_3_. Furthermore, due to the implementation of high pressure, the values of *B*, *G*, and *E* also rises, signifying that the implementation of compressive stress enhances the hardness, stiffness, and greater resistance to Sr_3_BCl_3_ (B = As, Sb) against tensile deformation.

**Table 6:** Calculate bulk modulus *B* (GPa), shear modulus *G* (GPa), young modulus *E* (GPa), Pugh's ratio *B/G*_H_, and Poisson ratio *ν*, Kleinman parameter ζ, and Zener anisotropy factor *A* at variant pressure 0–25 GPa.

Pressure (GPa)	Compound	*B*	*Y*	*G*	*B/G*	*ν*	ζ	*A*
0	Sr_3_AsCl_3_	31.71	51.03	20.73	1.529	0.231	0.284	0.467
	Sr_3_SbCl_3_	27.38	43.35	17.55	1.560	0.234	0.279	0.441
5	Sr_3_AsCl_3_	52.27	65.90	25.70	2.033	0.282	0.269	0.256
	Sr_3_SbCl_3_	42.29	57.30	22.24	1.901	0.289	0.262	0.226
10	Sr_3_AsCl_3_	71.08	75.38	28.87	2.462	0.305	0.262	0.164
	Sr_3_SbCl_3_	65.31	65.99	25.22	2.589	0.308	0.255	0.139
15	Sr_3_AsCl_3_	88.91	81.88	31.10	2.858	0.316	0.259	0.109
	Sr_3_SbCl_3_	82.30	71.88	27.30	3.014	0.317	0.251	0.088
20	Sr_3_AsCl_3_	105.94	86.31	32.68	3.241	0.320	0.256	0.072
	Sr_3_SbCl_3_	98.39	75.72	28.74	3.423	0.318	0.249	0.053
25	Sr_3_AsCl_3_	122.18	88.97	33.72	3.623	0.320	0.255	0.043
	Sr_3_SbCl_3_	113.28	77.67	29.56	3.832	0.314	0.247	0.027

**Fig. 11:**
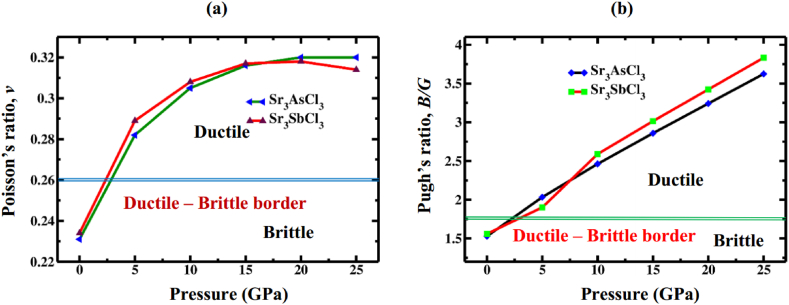
The variation in (a) Poisson's ration, and (b) Pugh's ratio as a function of applied pressure.

The Cauchy pressure (*C*_P_), Pugh's ratio (*B*/*G*), and Poisson's ratio (*ν*) are parameters that help us to determine the ductile or brittle characteristics of a material. Cauchy's pressure, expressed as the difference between the elastic constants *C*_12_ and *C*_44_, is regarded as an indicator of ductility: when the pressure is positive (negative), it suggests the material's ductile (brittle) nature [69]. Table 5 illustrates that Sr_3_AsCl_3_ and Sr_3_SbCl_3_ exhibit brittleness under ambient pressure conditions but indicates ductility under stress, as inferred from sign of *C*_P_. These findings indicate that Sr_3_BCl_3_ (B = As, Sb) becomes notably more ductile under pressure, with the sole exception being at ambient pressure. When Pugh's ratio and Poisson's ratio reaches the cutoff value of 1.75 and 0.26, respectively, referred to as the brittle/ductile borderline, it serves to differentiate the material as either brittle or ductile in its crystalline form. A material is considered ductile if both the ratios *B/G* and *ν* exceed their threshold value; if not, it is identified as brittle. At ground state, the determined *B/G* (*ν*) values for Sr_3_AsCl_3_ and Sr_3_SbCl_3_ are 1.529 (0.231) and 1.560 (0.234), respectively, validating the brittle characteristic of both materials. Fig. 11a & b clearly indicate that the Sr_3_BCl_3_ (B = As, Sb) materials surpassed the cutoff values of Pugh's ratio and Poisson's ratio under pressure, providing evidence of their high ductility level. Therefore, the calculated values of *B/G* and *ν* for both materials align with the *C*_P_ value, affirming their brittleness at 0 GPa and transitioning to ductile behavior as pressure increases. A Poisson's ratio between 0.25 and 0.50 categorizes materials as central force crystals, while those outside this range are non-central force crystals, stabilized by non-central forces [70]. According to Table 6, all four perovskites exhibit Poisson's ratios within the specified range (0.25 − 0.50) and are stabilized by central forces [71].

The Kleinman parameter (ζ), devoid of any units, spans from zero to one. This index indicates the relative ease with which bonds can be stretched as compared to others [72]. The Kleinman parameter can be found by:

(6) $$\zeta =\frac{C_{11}+8C_{12}}{{7C}_{11}+2C_{12}}$$

When bond stretching is minimized, ζ tends toward 1, while bond bending is minimized as ζ goes near 0. As depicted in Table 6, the determined value suggests that Sr_3_BCl_3_ (B = As, Sb) displays a stretching type of bonding under both the ground state and pressure-induced state.

The anisotropy factor (*A*) characterizes the directional dependence of a compound. It is computed through the Zener anisotropy factor formulae [73] for cubic structures.

(7) $$A=\frac{2C_{44}}{C_{11}-C_{12}}$$

**Fig. 12:**
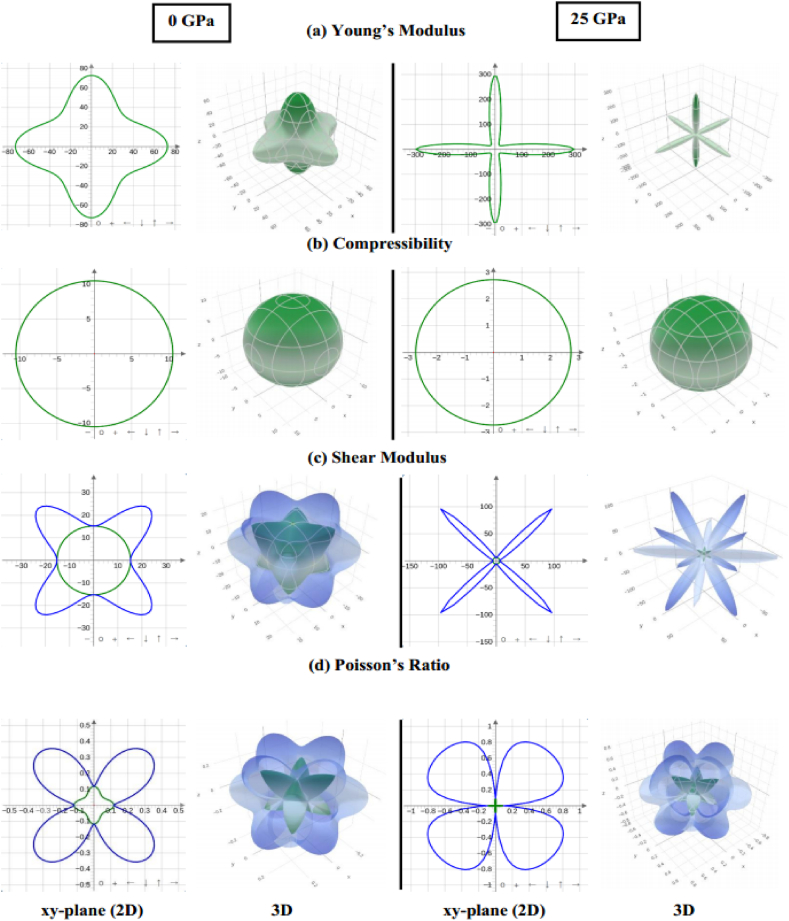
The anisotropic 2D and 3D visualization of (a) Young's modulus, (b) Compressibility, (c) shear modulus, and (d) poisson's ratio of Sr_3_AsCl_3_ at 0 GPa and 25 GPa pressure.

**Fig. 13:**
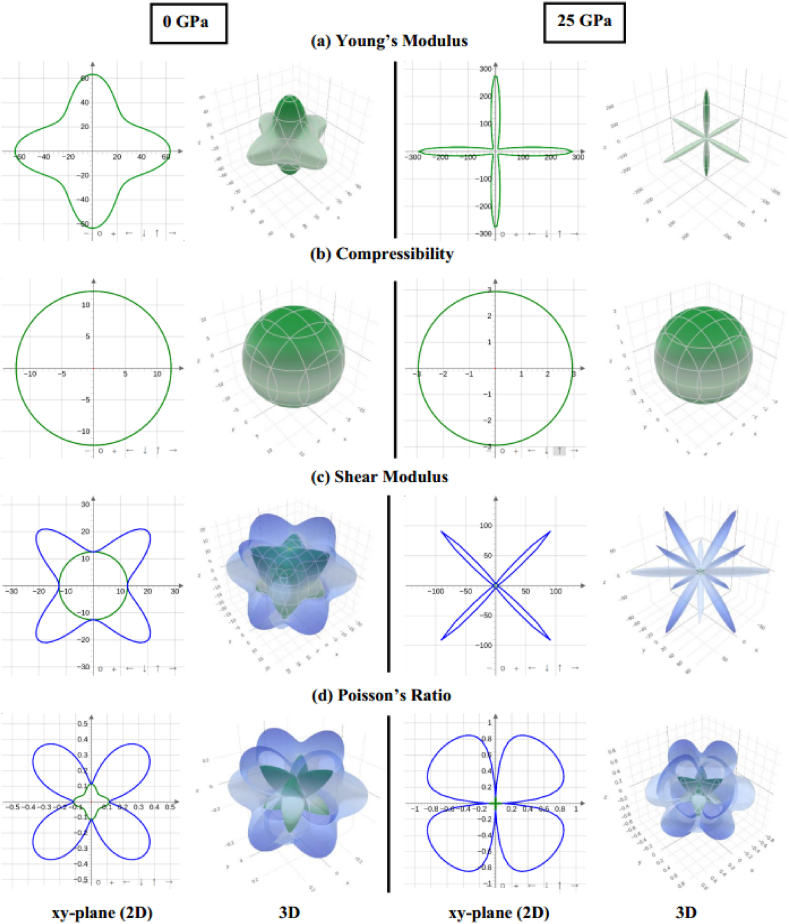
The anisotropic 2D and 3D visualization of (a) Young's modulus, (b) Compressibility, (c) shear modulus, and (d) poisson's ratio of Sr_3_SbCl_3_ at 0 GPa and 25 GPa pressure.

The calculated value of *A* deviates from 1 (Table 6), signifying an anisotropic nature in Sr_3_BCl_3_ (B = As, Sb), and this characteristic is improved with increasing pressure. The ELATE tool [74] was utilized to generate 2D and 3D visual representations illustrating the directional dependence of the Young's modulus (*E)*, shear modulus (*G*)*,* linear compressibility (β) and Poisson's ratio (*v)* under 0 and 25 GPa pressure conditions for Sr_3_AsCl_3_ and Sr_3_SbCl_3_, as dispayed in Fig. 12, Fig. 13. Spherical plots in three dimensions (3D) indicate isotropy, whereas non-spherical plots suggest anisotropy. Three-dimensional contour plots, which are non-spherical, indicate the existence of elastic anisotropy in all directions within the Sr_3_BCl_3_ (B = As, Sb) perovskites being investigated. For Sr_3_BCl_3_ (B = As, Sb), the parameters mentioned above exhibited similar responses across the xy, xz, and yz planes.

### 3.5 Thermal properties

The Debye temperature ($\Theta_{D}$) is a vital indicator of the bonding strength among atoms within crystals, representing the peak frequency of lattice vibrations. One widely used approach to determine $\Theta_{D}$ involves utilizing elastic constants, as $\Theta_{D}$ can be determined from average sound velocity ($v_{m}$). To determine $v_{m}$ of a material, the following equation is utilized [75,76]:

(8) $$v_{m}={\left[\frac{1}{3}\left(\frac{2}{{v_{s}}^{3}}+\frac{1}{{v_{l}}^{3}}\right)\right]}^{-1/3}$$

In this context, $v_{s}$ and $v_{l}$ denote the shear and longitudinal sound velocities, respectively. These velocities can be calculated by using the values of *B* and *G* in the following equation [75,76]:

(9) $$v_{s}=\sqrt{\frac{G}{\rho }}$$

(10) $$v_{l}=\sqrt{\frac{3B+4G}{3\rho }}$$

(11) $$\Theta_{D}=\frac{h}{k_{B}}{\left[\frac{3n}{4\pi }\left(\frac{N_{A}\rho }{M}\right)\right]}^{\frac{1}{3}}v_{m}$$

In the above equation h, $k_{B}$, $N_{A}$, n, M, ρ, $v_{m}$ are constants. Table 7 indicates that under ambient pressure, using the GGA-PBE functional, the Debye temperatures for Sr_3_AsCl_3_ and Sr_3_SbCl_3_ are 261.8 K and 232.9 K, respectively. The Debye temperature calculation for Sr_3_AsCl_3_ exceeds that of Sr_3_SbCl_3_, suggesting a higher melting point and stronger atomic bonding within the Sr_3_AsCl_3_ structure compared to Sr_3_SbCl_3_. Furthermore, the value of $\Theta_{D}$ of both substances rise as hydrostatic pressure increases. Previous studies [77,78] have also indicated that pressure has the potential to increase the Debye temperature. $\Theta_{D}$ correlates directly with the heat capacity. The increase in $\Theta_{D}$ influences the specific heat capacity of the examined materials, causing it to rise as the temperature increases. The increase in specific heat serves as an indicator of the thermodynamic stability of the materials under study [79]. The melting temperature refers to how a material transitions between different chemical phases as temperature varies. The evaluation of industrial material applications also considers their melting temperature. For the cubic system, the melting temperature is calculated by Ref. [80]:

(12) $$T_{m}=553+5.91C_{11}$$

**Table 7:** Pressure dependence of shear sound velocity $v_{s}$ (m/s), longitudinal sound velocity $v_{l}$ (m/s), average wave velocity $v_{m}$ (m/s), Debye temperatures $\Theta_{D}$ (K) and melting temperature T_m_(K) for the Sr_3_BCl_3_ (B = As and Sb).

Pressure (GPa)		Sr_3_AsCl_3_					Sr_3_SbCl_3_			
	$v_{s}$	$v_{l}$	$v_{m}$	$\Theta_{D}$	*T*_m_	$v_{s}$	$v_{l}$	$v_{m}$	$\Theta_{D}$	*T*_m_
0	2538.2	4294.8	2812.0	261.8	996.6	2331.0	3965.2	2583.7	232.9	938.8
5	2659.1	4879.5	2966.0	287.5	1302.6	2449.0	4404.8	2727.5	257.4	1238.3
10	2705.0	5270.0	3029.7	301.9	1582.8	2493.5	4938.8	2795.7	271.9	1509.9
15	2720.5	5570.3	3056.0	310.9	1848.1	2507.3	5228.2	2819.3	280.5	1765.6
20	2717.7	5813.1	3059.8	316.7	2101.7	2502.0	5456.9	2819.5	285.8	2007.7
25	2700.5	6012.4	3046.0	320.0	2343.4	2477.9	5631.8	2797.3	288.0	2233.7

The melting temperatures of both compounds studied increased as pressure rose (Table 7), rendering them suitable for various industries such as metallurgy, manufacturing, and aerospace. Their capacity to endure high temperatures without undergoing phase transitions enhances their applicability.

### 3.6 Optical properties

The optical parameters (absorption, reflectivity, refractive index, extinction coefficient, conductivity, loss function, and the real and imaginary parts of the dielectric function) of Sr_3_BCl_3_ (B = As, Sb) perovskite are computed in the spectrum of photon energies spanning between 0 and 20 eV to find the possible utilization of these materials in optoelectronic applications. The interplay at the material's surface and incident electrons or photons can be explained through the dielectric function ε(ω) as,

(13) $$\varepsilon \left(\omega \right)=\varepsilon_{1}\left(\omega \right)+i\varepsilon_{2}\left(\omega \right)$$

Here, $\varepsilon_{1}\left(\omega \right)$ and $\varepsilon_{2}\left(\omega \right)$ signifies the real and imaginary portion of the dielectric function ε(ω), respectively. The Kramer-Kronig relation is employed for deducing the real component $\varepsilon_{1}\left(\omega \right)$ of the dielectric function, expressed as follows [81]:

(14) $$\varepsilon_{1}\left(\omega \right)=1+\frac{2}{\pi }P\int_{0}^{\infty }\frac{\omega^{\prime \varepsilon_{2}}\left(\omega^{\prime }\right)}{\omega^{\prime 2}-\omega^{2}}d\omega^{\prime }$$

The real transition from occupied to unoccupied electronic states can be utilized for computing the imaginary dielectric constant $\varepsilon_{2}\left(\omega \right)$, expressed as:

(15) $$\varepsilon_{2}\left(\omega \right)=\frac{2e^{2}\pi }{\Omega \varepsilon_{\circ }}\sum_{K,V,C}\left|{<\psi }_{k}^{c}\right|\hat{U}|.\vec{r}|\psi_{k}^{v}|>{|}^{2}\delta \left(E_{k}^{c}+E_{k}^{v}-E\right)$$

Various optical parameters, including the absorption coefficient *α* (ω), reflectivity *R* (ω), refractive index *n* (ω), extinction coefficient *k* (ω), optical conductivity σ(ω) and loss function *L* (ω), can be derived from the complex dielectric function utilizing the following equations [82,83]:

(16) $$\alpha \left(\omega \right)=\frac{4\pi k}{\lambda }$$

(17) $$R\left(\omega \right)=\frac{{\left(n-1\right)}^{2}+k^{2}}{{\left(n+1\right)}^{2}+k^{2}}$$

(18) $$n\left(\omega \right)={\left[\frac{\left\{\sqrt{\varepsilon_{1}^{2}\left(\omega \right)}+\varepsilon_{2}^{2}\left(\omega \right)\right\}-\varepsilon_{1}\left(\omega \right)}{2}\right]}^{\frac{1}{2}}$$

(19) $$k\left(\omega \right)={\left[\frac{\{{\varepsilon_{1}^{2}\left(\omega \right)+\varepsilon_{2}^{2}\left(\omega \right)\}}^{\frac{1}{2}}-\varepsilon_{1}\left(\omega \right)}{2}\right]}^{\frac{1}{2}}$$

(20) $$\sigma \;\left(\omega \right)=\frac{\alpha \left(\omega \right)n\left(\omega \right)c}{4\pi }$$

(21) $$L\left(\omega \right)\frac{\varepsilon_{2}\left(\omega \right)}{\varepsilon_{1}^{2}\left(\omega \right)+\varepsilon_{2}^{2}\left(\omega \right)\;}$$

**Fig. 14:**
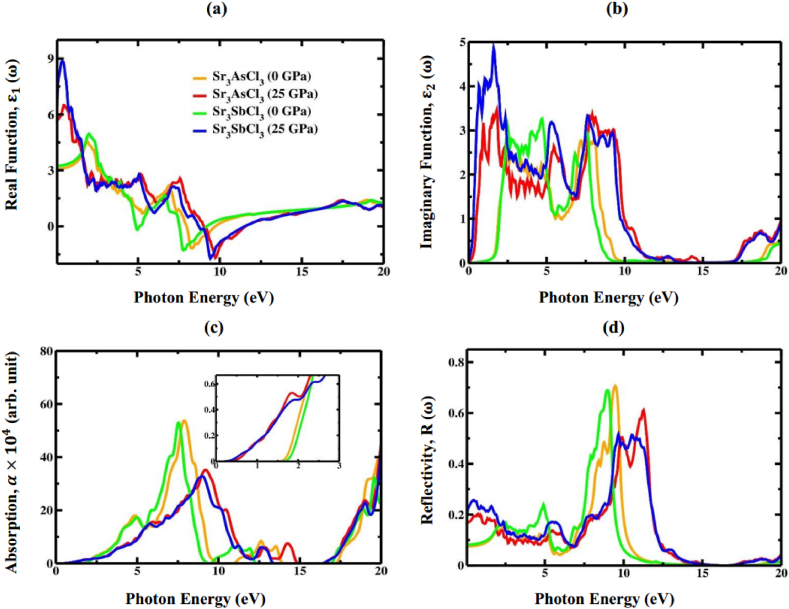
The calculated (a) real part, (b) imaginary part, (c) absorption, and (d) reflectivity of Sr_3_BCl_3_ (B = As, Sb) at 0 and 25 GPa pressure.

In Fig. 14a, the pressure-dependent $\varepsilon_{1}\left(\omega \right)$ is presented for both investigated materials in the range of photon energies up to 20 eV. It is evident that as the pressure rises from 0 to 25 GPa, the static dielectric value $\varepsilon_{1}\left(0\right)$ increase (Fig. 14a). This is attributed to a reduced rate of charge recombination, rendering these compounds more suitable for application in optoelectronic devices. At around 7 eV–10 eV of input photon energy, both halide perovskites attain negative peak values, signifying complete reflection within these energy intervals. Furthermore, the figure illustrates that the Sr_3_BCl_3_ (B = As, Sb) compound demonstrates the highest $\varepsilon_{1}$ in the visible range under pressure. Due to an inverse correlation between the band gap and $\varepsilon_{1}\left(0\right)$, a higher energy gap leads to a smaller $\varepsilon_{1}\left(0\right)$ value. This relationship, in accordance with the Penn model [84], can be elucidated by the following equation:

(22) $$\varepsilon_{1}\left(0\right)\approx 1+{\left(\frac{{\hbar \omega }_{p}}{E_{g}}\right)}^{2}$$

Recently, researchers [[85], [86], [87]] have conducted numerous investigations demonstrating that the band gap and static dielectric value are inversely related, with increasing pressure leading to a decrease in the band gap and consequently reducing the static dielectric value, in line with the Penn Model. These findings strongly support the conclusions drawn in this study.

The imaginary part, $\varepsilon_{2}\left(\omega \right)\text{,}$ signifies the attenuation of light as it moves through a substance (Fig. 14b). $\varepsilon_{2}\left(\omega \right)$ is closely associated with the band gap of a substance and its optical absorption. $\varepsilon_{2}\left(\omega \right)$ contributes to the understanding of the bandgap, this is important for the energy involved in inter-band transitions near the *E*_F_ [88]. Fig. 14b reveals that for both compounds, $\varepsilon_{2}$ initiates at the corresponding fundamental band gap for each pressure and as pressure increases, the curve shift towards lowers value of photon energy. The apparent pattern in the imaginary component aligns with electronic band structure's behavior (Fig. 4, Fig. 5), indicating a decrease in the bandgap with increasing applied pressure. Optical absorption happens through the shifting of charges from the valence band maximum (As-4p (Sb-5p) to the conduction band minimum (Sr-4p). Most significantly, the $\varepsilon_{2}$ of Sr_3_AsCl_3_ and Sr_3_SbCl_3_ experiences a substantial increase in the visible spectrum due to the increase in pressure from 0 to 25 GPa. Hence, these selected materials exhibit significant potential in optoelectronic applications. The dielectric constant of a material dictates its ability to retain the electrical energy within an electric field [89]. In comparison to the non-pressurized configuration, as shown in Fig. 14a and b, pressure leads to increased real and imaginary parts of ε(ω).

Understanding the light absorption capability of a material is pivotal for determining its potential usefulness in optical devices. The absorption coefficient α(ω) signifies the decline in light intensity over a specific distance. The absorption coefficient α(ω) for both compound follows the same trends as $\varepsilon_{2}\left(\omega \right)$, as depicted in Fig. 14c. Upon closer examination of Fig. 14c, it becomes evident that the onset of absorption does not occur at 0 eV. At ambient pressure, the absorption coefficient α(ω) begins at the band gap as a result of absorbing ultraviolet radiation. It's widely recognized that solar cells and other photovoltaic applications greatly benefit from the initial peak in absorption. We have found that within the visible spectrum of Sr_3_BCl_3_ (B = As, Sb), there's a notable increase in light absorption as pressure rises compared to when it's at ambient levels. This underscores the critical importance of pressure effects on both materials for enhancing absorption in solar cells and other optoelectronic devices. Furthermore, Fig. 14c illustrate a noticeable shift in the absorption edge of both materials in the direction of lower energy area (red shift) under applied compressive stress. Schwarz et al. [90] also experimentally reported the red shift in absorption of the perovskite terminal, thus validating the accuracy of this present work. Under stress, Sr_3_BCl_3_ (B = As, Sb) exhibits a wider range of light absorption compared to its non-stressed counterparts, making it an appealing choice for use in photovoltaic applications. As both materials show a shift of their absorption maxima towards the UV spectrum as placed under pressure, they are excellent candidates for the development of medical equipment disinfection devices. So, it can be concluded that by imposing different hydrostatic pressure to the Sr_3_BCl_3_ (B = As, Sb) perovskite compound, there is a potential to alter the absorption spectral area, offering a way to adjust its optical characteristics.

The surface characteristics of Sr_3_BCl_3_ (B = As, Sb) can be investigated by assessing the light reflection from the material's surface. Fig. 14d represent the reflectivity spectrum of Sr_3_BCl_3_ (B = As, Sb) material under pressure conditions, encompassing the incident photon energies up to 20 eV. The graph shows that the highest reflectivity occurs when the value of $\varepsilon_{1}\left(\omega \right)$ is at its lowest or negative. As the external pressure increases, all the computed curves of *R* (ω) shift in the direction of high energy area. This indicates that the reflectivity of Sr_3_BCl_3_ (B = As, Sb) can be adjusted by applying external pressure. Furthermore, in the ultraviolet (UV) range, both materials exhibit maximum optical reflectivity, suggesting that Sr_3_BCl_3_ (B = As, Sb) can effectively function as a reflector throughout the UV light spectrum.

**Fig. 15:**
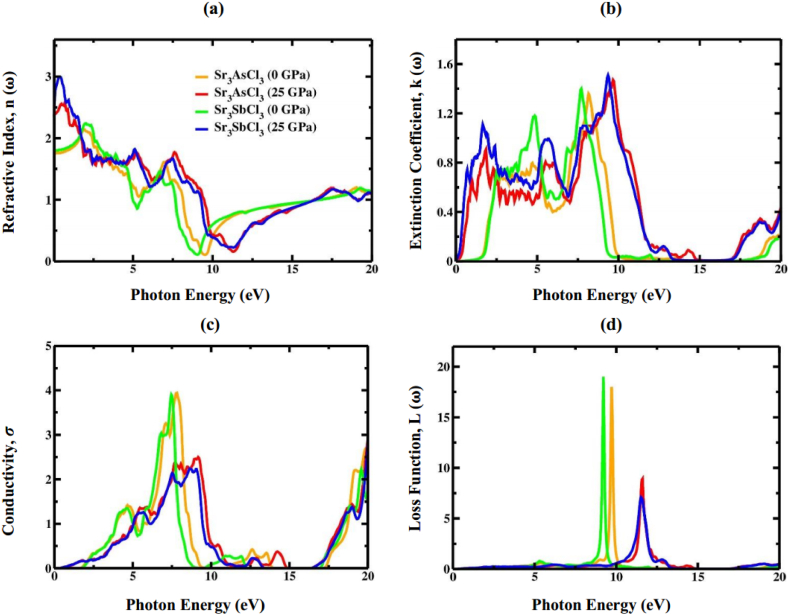
The calculated (a) refractive index, (b) extinction coefficient, (c) conductivity, and (d) loss function of Sr_3_BCl_3_ (B = As, Sb) at 0 and 25 GPa pressure.

The refractive index *n* (ω) can indicate the connection between the transparency and spectral radiation characteristics of the material. Fig. 15a, which illustrates the refractive index, closely mirrors the real component of ε(ω), as it is derived from $\varepsilon_{1}\left(\omega \right)$. At zero frequency of the applied field, the pattern of *n* (ω) mimics that of the real part of $\varepsilon_{1}\left(\omega \right)$ and is linked to it through the equation $n^{2}-k^{2}$ = $\varepsilon_{1}\left(0\right)$. Furthermore, the refractive index at zero frequency, denoted as *n* (0), and the static dielectric value $\varepsilon_{1}\left(0\right)$ precisely meet the requirement $n_{0}^{2}=$ $\varepsilon_{1}\left(0\right)$, as depicted in Fig. 14, Fig. 15a. When the incident photon energy falls within the range of 8–17 eV, the refractive index is below 1, indicating that within this range, the wave propagates faster in the Sr_3_AsCl_3_ and Sr_3_SbCl_3_ crystal than in a vacuum. Within this area, the group velocity (*V*_g_ = *c*/*n*) of incident radiation exceeds *c* because *n* (ω) has a value below unity [91,92]. As pressure increases, the rise in static refractive index values ($n_{0}$) suggests that these two materials can be effectively utilized for photonic applications [93]. The computed extinction coefficient *k* (ω) at 0 GPa and 25 GPa of applied pressure is shown in Fig. 15b, demonstrating a trend behavior to $\varepsilon_{2}\left(\omega \right)$.

Another crucial factor in investigating the optical properties of a material is the optical conductivity, denoted as σ(ω). Optical conductivity is another manifestation of photoconductivity. Both the photoconductivity and electrical conductivity increase as a result of greater absorption of photons. Fig. 15c depicts the optical conductivity σ(ω) of Sr_3_BCl_3_ (B = As, Sb) at pressures of 0 GPa and 25 GPa. σ(ω) has a direct correlation with the absorption coefficient and provides insight into the conduction of carriers following the absorption of photons. The optical conductivity for both materials increase at 25 GPa pressure, which stems from heightened absorption (Fig. 15c). The electron loss function (ELF), L(ω) is an optical parameter that quantifies the amount of energy that is dissipated by a fast-moving electron as it traverses a material. This optical parameter encompasses the examination of a material's reaction to light. *L* (ω) can be calculated utilizing the following equation:

(23) $$L\;\left(\omega \right)=j\;\left(-\frac{1}{\varepsilon \left(\omega \right)}\right)$$

In Fig. 15d, the pressure-dependent ELF for Sr_3_BCl_3_ (B = As, Sb) is presented. The plots of the ELF for both materials exhibited peaks, signifying a reduction in energy when incident photon's energy surpassed the compound's electronic bandgap. We observed that in both materials, most of the energy loss happens within the UV region because the photon energy exceeds that of the band gap energy. No dispersion was observed below the energy of the bandgap for both Sr_3_AsCl_3_ and Sr_3_SbCl_3_ material. Furthermore, ELF is associated with the plasma frequency (ω_p_). At ambient pressure, it was noted that in the case of Sr_3_AsCl_3_, the peak loss function is 9.71 eV, while for Sr_3_SbCl_3_, it is 9.21 eV. These discoveries offer rough estimates for ω_p_ of the Sr_3_BCl_3_ (B = As, Sb) materials. Beyond the plasma frequency, both materials become transparent at higher frequencies, meaning that the electrons within them cannot absorb the energy from the incoming light. The applied pressure causes the ELF structures to move toward lower energy levels while reducing the peak height for both compounds.

## 4 Conclusions

First principles DFT-based simulations were employed to investigate the physical properties of the non-toxic perovskite compounds Sr_3_BCl_3_ (B = As, Sb) under varying pressures up to 25 GPa. As pressure levels increase, there is an observed decreasing trend in the lattice parameter, volume, and bond length. It encompasses various physical properties, including structural, elastic, mechanical, Debye temperatures, electronic, and optical characteristics. As pressure increased, the lattice constants and unit cell volume decreased due to the reduced interatomic distances. Through the examination of electronic behavior, it is evident that the implementation of pressure has the capacity to alter the direct band gap of Sr_3_AsCl_3_ and Sr_3_SbCl_3_. The shift of Sr-4p states toward the Fermi level with increasing pressure is attributed to the observed change in electronic behavior. Furthermore, the effective masses of electrons and holes in Sr_3_BCl_3_ (B = As, Sb) perovskites decrease gradually under applied hydrostatic pressure. Effective mass values below 1.5*m*_0_ for Sr_3_BCl_3_ (B = As, Sb) compounds indicate a high mobility for the charge carriers. The calculated elastic constants consistently meet the Born stability criteria, affirming the mechanical stability of the studied compounds across all applied pressure ranges. Under normal atmospheric pressure, both materials exhibit brittleness, which transitions to ductility as pressure increases. The investigation of optical properties suggests that novel Sr_3_BCl_3_ (B = As, Sb) perovskite compounds have multifunctional applications in optoelectronic technologies. Finally, it is speculated that the outcomes of this research will motivate the researcher to carry out an experimental synthesis on novel Sr_3_BCl_3_ (B = As, Sb) perovskites, with the goal of achieving success in their applications for thermoelectric and optoelectronic purposes.

## CRediT authorship contribution statement

**Asif Hosen:** Writing – review & editing, Writing – original draft, Visualization, Validation, Methodology, Investigation, Data curation, Conceptualization.

## Declaration of competing interest

The authors declare that they have no known competing financial interests or personal relationships that could have appeared to influence the work reported in this paper.
